# Refractory Clinical Course of Acute Myeloid Leukemia With t(8; 10; 21)(q22; q22; q22.1): A Case Report and Literature Review of This Variant Form

**DOI:** 10.7759/cureus.100882

**Published:** 2026-01-05

**Authors:** Yuta Yamada, Toshiaki Nagaie, Rika Tomimasu, Masaharu Miyahara, Noriyasu Fukushima

**Affiliations:** 1 Department of Internal Medicine, Karatsu Red Cross Hospital, Karatsu, JPN

**Keywords:** acute myeloid leukemia, obesity, runx1::runx1t1, three-way translocation, translocation chromosomes 8 and 21

## Abstract

Acute myeloid leukemia (AML) with t(8;21)(q22;q22.1)/RUNX1::RUNX1T1 is usually associated with a good prognosis and is categorized as a favorable-risk group in the European Leukemia Net recommendations. A variant form involving a three-way translocation is rarely observed in this AML subtype, and the prognostic impact of this form remains unclear. A 43-year-old man presented with anemia and thrombocytopenia. He had diabetes mellitus and class III obesity according to the WHO criteria. After being diagnosed with AML with t(8;10;21)(q22;q22;q22.1), he received standard induction therapy consisting of cytarabine for seven days and idarubicin for three days, but failed to achieve remission. Reinduction and salvage therapies were also ineffective. This variant form may be associated with a lower likelihood of achieving complete remission (CR) after initial induction therapy. Failure to achieve CR after initial induction therapy in AML is an adverse prognostic factor; therefore, the presence of t(8;21)/RUNX1::RUNX1T1 with three-way translocations may warrant consideration of hematopoietic stem cell transplantation at an earlier stage in the treatment course.

## Introduction

Acute myeloid leukemia (AML) with t(8;21)(q22;q22.1)/RUNX1::RUNX1T1 accounts for approximately 7% of all AML cases and is generally associated with a favorable prognosis, compared to intermediate and adverse risk groups based on specific genetic mutations and chromosomal abnormalities in European Leukemia Net (ELN) stratification. The t(8;21)(q22;q22.1) translocation is a leukemogenic alteration that results in the formation of the chimeric fusion gene RUNX1::RUNX1T1, which acts as a dominant repressor of RUNX1-mediated hematopoietic gene expression, thereby disrupting normal hematopoietic differentiation and promoting a preleukemic state [1]. Variant translocations, most of which are complex three-way translocations, occur in approximately 3%-4% of AML cases with t(8;21) [2,3]. Certain chromosomal abnormalities in AML involving RUNX1::RUNX1T1 have been shown to affect clinical outcomes; specifically, deletions of chromosome arm 9q are associated with a reduced likelihood of achieving complete remission (CR), whereas gain of chromosome 4 is associated with an inferior cumulative incidence of relapse and overall survival [4]. On the other hand, the clinical and hematological characteristics of AML involving RUNX1::RUNX1T1 with three-way translocations remain poorly understood owing to the absence of comprehensive studies. Genetic mutations in AML have prognostic significance: mutations such as Nucleophosmin 1 and biallelic enhancer binding protein-alpha generally indicate a favorable prognosis, whereas FLT3 mutation in AML with normal karyotype and KIT mutations in AML involving RUNX1::RUNX1T1 are associated with poor prognosis and lower remission rates. The prognostic impact of this variant chromosomal form, as well as the relationship among these genetic mutations, has not yet been explored. Herein, we describe a complex three-way translocation, t(8;10;21)(q22;q22;q22.1), in a case of AML with RUNX1::RUNX1T1, and provide a review of previously reported cases of AML with similar three-way translocations in the literature.

## Case presentation

**Figure 1:**
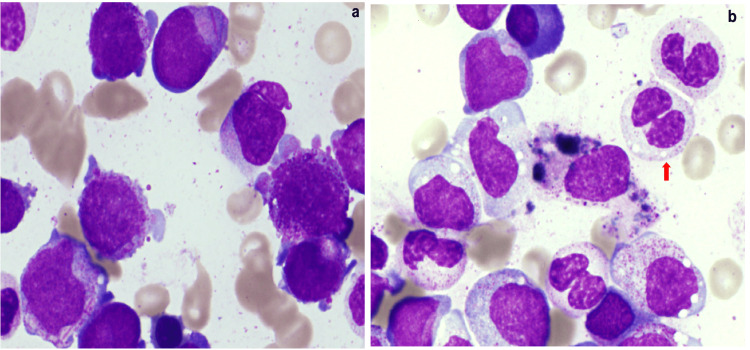
Bone marrow morphology (a,b) Myeloblasts contained Auer rods and some azurophilic granules in the basophilic cytoplasm. The pseudo-Pelger-Huët anomaly (red arrow) was observed in mature neutrophils (May-Grünwald-Giemsa stain 400×)

A 43-year-old man was admitted due to anemia and thrombocytopenia. He had diabetes mellitus and was markedly obese, with a body mass index of 41.9. Physical examination was unremarkable except for pallor of the conjunctivae. Laboratory tests revealed a hemoglobin concentration of 71 g/L and a platelet count of 15 × 10⁹/L. The white blood cell (WBC) count was 7.3 × 10⁹/L, with 14% myeloblasts (Table 1). Bone marrow examination revealed marked hypercellular marrow with 28.8% myeloblasts, which contained Auer rods and some azurophilic granules in the basophilic cytoplasm (Figures 1a, 1b).

**Table 1:** Laboratory findings at the admission Myeloblasts were observed in peripheral blood. Anemia and thrombocytopenia were presented. WT-1 was 5,200 copy/μg RNA WBC: white blood cell; PT: prothrombin time; APTT: activated partial thromboplastin time; AST: aspartate aminotransferase; ALT: alanine aminotransferase; FDP: fibrin-degradation product; LDH: lactate dehydrogenase; RBC: red blood cell; ALP: alkaline phosphatase; TP: total protein; BUN: blood urea nitrogen; CRP: C-reactive protein; WT-1: Wilms tumor-1; Seg: segmented neutrophils; Lym: lymphocytes; Hb: hemoglobin; Ht: hematocrit; Plt: platelets; Fib: fibrinogen; T-bil: total bilirubin; Alb: albumin; Cr.: creatinine

Parameter	Values
WBC	7.3 × 10^9^/L
Myeloblast	14%
Seg	51%
Lym	35%
RBC	1.83 × 10^12^/L
Hb	71 g/L
Ht	20.2%
Plt	15 × 10^9^/L
PT	9.8 seconds
APTT	23.7 seconds
Fib	486 mg/dL
FDP	6.5 μg/mL
D-dimer	3.8 μg/mL
TP	7.7 g/dL
Alb	3.6 g/dL
BUN	12.1 mg/dL
Cr.	0.63 mg/dL
T-bil	0.46 mg/dL
AST	35 U/L
ALT	20 U/L
LDH	292 U/L
ALP	95 U/L
Na	138 mmol/L
K	3.7 mmol/L
Cl	103 mmol/L
CRP	1.51 mg/dL
WT-1	5,200 copy/μg RNA

**Figure 2:**
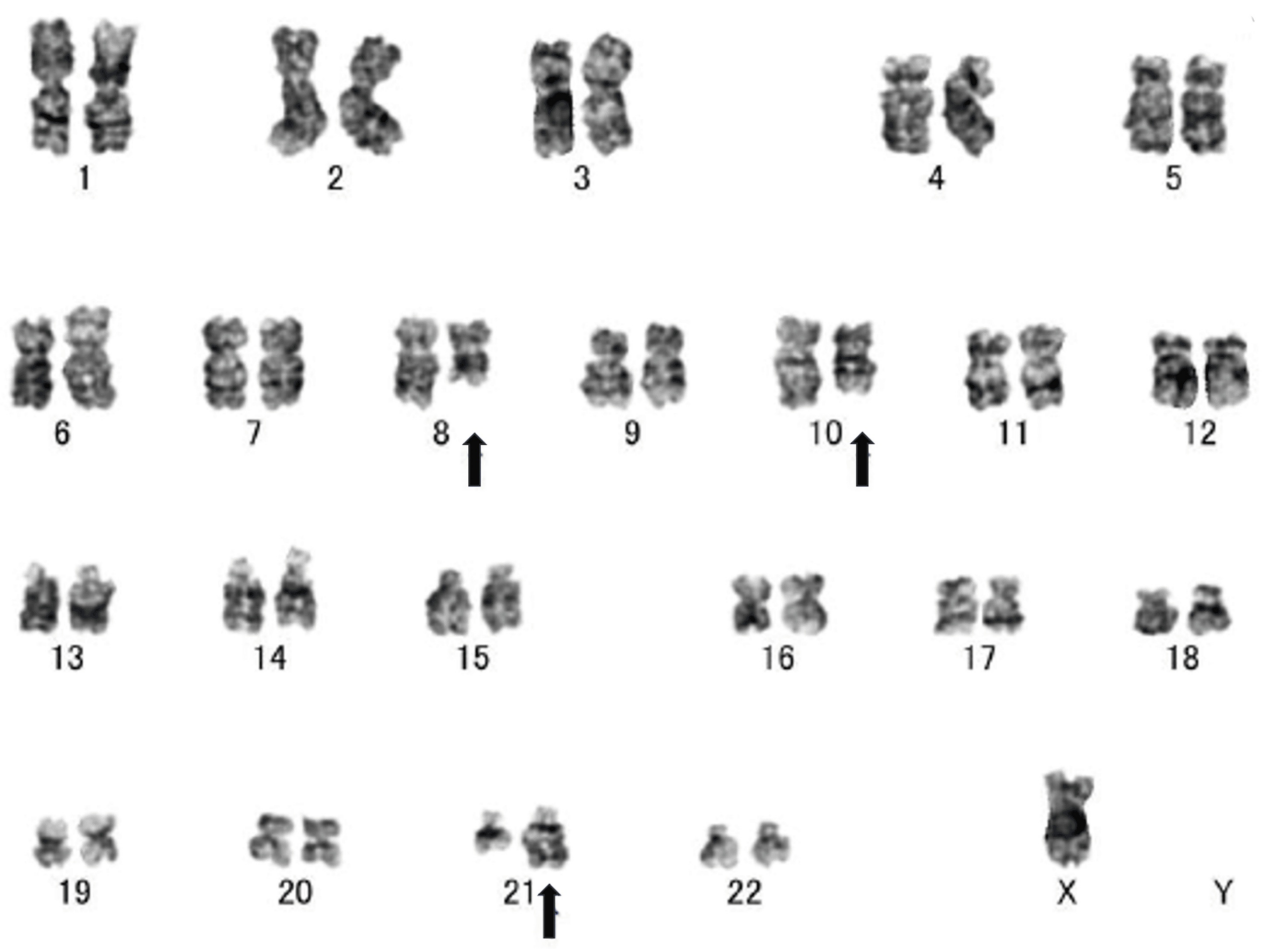
Chromosomal analysis in bone marrow. G-banded cytogenetic analysis revealed 45,X,-Y,?t(8;10;21)(q22;q22;q22). Arrows show translocated chromosomes

The pseudo-Pelger-Huët anomaly was observed in mature neutrophils. The myeloblasts were positive for myeloperoxidase staining. Immunophenotypic analysis showed that the myeloblasts were positive for CD13, CD22, CD34, and HLA-DR by flow cytometry. CD117 and CD19 were weakly positive. Cytogenetic analysis revealed 45, X, -Y, ?t(8;10;21)(q22;q22;q22.1) in nine out of 20 cells (Figure 2). RUNX1::RUNX1T1 was 6.3 × 10⁴ copies/μg RNA, as measured by real-time quantitative reverse transcriptase-polymerase chain reaction. The patient was diagnosed with AML with RUNX1::RUNX1T1 fusion, according to the WHO 5th edition classification. No FLT3-Internal Tandem Duplication/Tyrosine Kinase Domain, NPM1, or KIT mutations were detected.

The patient received induction chemotherapy with cytarabine (100 mg/m² for seven days) and idarubicin (12 mg/m² for three days). Myeloblasts in the peripheral blood initially decreased but increased again on day 21. The rate of myeloblasts in bone marrow was 38.2% on day 28, and RUNX1::RUNX1T1 was 2.9 × 10⁴ copies/μg RNA. Reinduction therapy using the same regimen was administered, but failed to achieve remission. After receiving bridging therapy with venetoclax and azacitidine in preparation for allogeneic hematopoietic stem cell transplantation (HSCT), the patient was transferred to the another hospital. He received an allogeneic peripheral blood HSCT from a haploidentical donor and survived for 16 months after HSCT.

## Discussion

AML with t(8;21); RUNX1::RUNX1T1 is recognized as a distinct subtype. This subtype often occurs in young patients and is categorized as a favorable group in ELN stratification [5]. The t(8;21) variant translocation involving other chromosomes has been reported with a frequency of approximately 3%-4% [6]. Clinicopathological features of t(8;21); RUNX1::RUNX1T1 with three- or four-way translocation are less characterized than the standard translocation, and the prognostic impact of this cytogenetic variant is still unknown. We herein summarize previous reports that described the efficacy of induction therapy and outcomes in cases of t(8;21)/RUNX1::RUNX1T1 with three-way translocations.

We searched the PubMed database for case reports of AML with t(8;21)(q22;q22)/RUNX1::RUNX1T1 involving a three-way translocation, focusing on the efficacy of induction therapy and patient outcomes. Cases lacking information on induction chemotherapy regimens or treatment efficacy were excluded. We did not limit the language or publication-year restrictions. A total of 26 cases were identified and included in the analysis (Table 2) [3,7-27]. Because most reports lacked detailed information on transplantation status or long-term prognosis, these variables were not analyzed.

**Table 2:** Review of the previous AML cases that have t(8;21)/RUNX1::RUNX1T1 with three-way translocations IDA: idarubicin; HDAC: high-dose Ara-C; DNR: daunorubicin; VCR: vincristine; MIT: mitoxantrone; HU: hydroxyurea; Flu: fludarabine; MMC: mitomycin C; N.D.: not done; CR: complete remission; AML: acute myeloid leukemia CR was achieved; however, the number of chemotherapy cycles required is not described

No.	Age	Sex	Karyotype	Another partner chromosome of the three-way translocation	Regimen	Number of times to achieve remission	Reference
1	33	M	t(21;8;1)(q22;q22;q32)^*^	1	IDA+Ara-C	Once	[7]
2	49	M	t(1;21;8)(q25;q22;q22)^*^	1	Flu+Ara-C	Once	[3]
3	45	M	46,XY,t(1;21;8)(p36.1;q22;q22)	1	IDA+Ara-C	Once	[8]
4	51	M	45,X,-Y,t(2;21;8)(q13;q22;q22) 46,XY,t(5;9)(q15;q22)	2	IDA+Ara-C	Once	[9]
5	55	M	45,X,-Y,t(2;21;8)(q36;q22;q22)	2	IDA+Ara-C→HDAC	Twice	[10]
6	5	M	46,XY,der(7)t(4;7)(q27;q32),t(8;21;2)(q22;q22;p13),del(9)(q22)	2	Ara-C+VP-16	CR	[11]
7	25	F	46,XX,t(3;21;8)(q21;q22;q22)	3	DNR+Ara-C+VCR→DNR+HDAC	Fail	[12]
8	9	F	46,XX,t(5;8;21) (q33;q22;q22)	5	Ara-C＋VP-16＋MIT	Once	[13]
9	25	F	46, XX,t(6;21;8)(p23;q22;q22)	6	IDA+Ara-C	Once	[14]
10	37	F	46,XX,t(6;21;8)(p21;q22;q22). 47,XX,+4,t(6;21;8)(p21;q22;q22)	6	DNR+Ara-C+MIT+VP-16	CR	[15]
11	57	M	46,XY,t(8;10;21)(q22;q25;q22). 45,X,-Y,t(8;10;21). 46,XY	10	N.D.	CR	[16]
12	11	F	t(8;10;21)(q22;q24;q22)^*^	10	DNR+Ara-C+VP-16	Once	[17]
13	63	F	46,XX,t(8;10;21)(q22;p14;q22)	10	HU→DNR+Ara-C→DNR+Ara-C	Twice	[18]
14	62	M	46,XY,t(8;11;21)(q22;q24;q22)	11	IDA+Ara-C→DNR+Ara-C	Twice	[19]
15	27	F	t(8;11;21)(q22;q13;q22)^*^	11	Flu+Ara-C	Once	[3]
16	56	F	46,XX,t(8;12;21)(q22;p11;q22),del(9)(q?), 46,XX	12	IDA+Ara-C	CR	[20]
17	51	M	45,X,-Y,t(8;12;21)(q22.1;q24.1;q22.1)	12	Behenoyl Ara-C+DNR+6-MP+PSL	CR	[21]
18	33	F	46,XX,t(8;13;21)(q22:q14;q22)	13	DNR+Ara-C	Once	[22]
19	62	F	46,XX,t(8;14;21)(q22;q13;q22),t(15;21)(q15;p11)	14	IDA+Ara-C	Once	[23]
20	25	M	46,XY,t(8;14;21)(q22;q32;q22). 45,X,-Y,t((8;14;21)	14	N.D.	CR	[16]
21	38	M	45,X,Y,del(3)(p25),t(8;21;14)(q22;q22;q24),der(19)t(3;19)(?p25;p13)	14	IDA+Ara-C→HDAC	Twice	[24]
22	49	F	46,XX,t(8;16;21)(q22;q12-13;q22)	16	N.D.	Twice	[16]
23	39	M	t(8;20;21)(q22;q13;q22)	20	DNR+Ara-C	CR	[25]
24	20	M	45,X,-Y,t(8;20;21)(q22;q13.2;q22)	20	N.D.	CR	[16]
25	25	F	46,XX,t(8;22;21)(q22;q12;q22). 45,X,sl,-X. 46,XX.	22	DNR+Ara-C	Once	[26]
26	30	M	45,X,t(X;21;8)(p22.1;q22;q22),-Y	X	MMC+Ara-C(3+7)	Once	[27]
Present case	43	M	45,X,-Y,?t(8;10;21)(q22;q22;q22.1)	10	IDA+Ara-C	Fail	-

The third chromosome involved in the three-way translocation, in addition to chromosomes 8 and 21, varied among the cases. Most patients received induction therapy consisting of a combination of an anthracycline and cytarabine. Approximately 20% of patients did not achieve CR with the first induction therapy, although the overall CR rate among all patients was 96%. Our patient was also ineffective in induction and reinduction therapy. Even in cases of three-way translocations without additional chromosomal abnormalities, the CR rate with initial induction therapy was 60%. In the Cancer and Leukemia Group B (CALGB) 8461 study, the CR rate by the first induction for patients of AML with t(8;21) was 89%, and secondary cytogenetics did not affect the CR rate [28]. The CR rate of AML with t(8;21)/RUNX1::RUNX1T1 with three-way translocations was inferior to the result of this study. This cytogenetic abnormality may adversely affect the CR.

Prognostic factors in AML include male sex, advanced age, high WBC count at diagnosis, secondary chromosomal abnormalities, failure to achieve CR after initial induction therapy, and genomic mutation, such as KIT mutation [28]. In CALGB 8491, non-White AML patients with t(8;21) and secondary chromosomal abnormalities exhibited inferior overall survival compared to those with t(8;21) alone; however, no significant difference in overall survival was observed among White patients [6]. Therefore, it is still controversial whether t(8;21)/RUNX1::RUNX1T1 with additional chromosomal abnormality affects overall survival. In the present case, we were unable to determine whether t(8;21)/RUNX1::RUNX1T1 with three-way translocations impacts overall survival, due to the absence of long-term observational studies and the limited availability of data, which currently consist only of case reports. Furthermore, most case reports have not reported the results of gene-mutation analyses, although the presence of a KIT mutation is known to adversely affect prognosis in AML with t(8;21)(q22;q22)/RUNX1::RUNX1T1. A KIT mutation was not detected in our patient, but his leukemia was refractory to both induction and salvage therapy. Further analysis with a larger number of cases is needed to clarify the relationship between gene mutations and three-way translocations.

Our patient was classified as having Class III obesity according to the WHO criteria. The relationship between obesity and prognosis in AML remains controversial. In the SWOG S1203 trial, Class III obesity was associated with worse overall survival after allogeneic stem cell transplantation [29]. On the other hand, the Eastern Cooperative Oncology Group American College of Radiology Imaging Network analysis found that obesity was not associated with inferior response or survival, even though obese patients received less than 90% of the intended daunorubicin dose [30]. Additionally, this study did not observe any association between obesity and the presence of somatic mutations such as FLT3, NPM1, or KIT [30]. Therefore, the reason our patient failed to achieve CR after both induction and reinduction therapy may not be related to obesity.

## Conclusions

AML with t(8;21)(q22;q22)/RUNX1::RUNX1T1 involving a three-way translocation may be associated with a lower likelihood of achieving CR following first induction therapy. Patients with these cytogenetic features are often classified as having primary refractory disease and are infrequently cured with conventional salvage therapy. Therefore, early evaluation for allogeneic HSCT is warranted. Although our analysis is limited to the case level, which restricts the interpretation of the results, the presence of t(8;21)/RUNX1::RUNX1T1 with a tripartite translocation may indicate that HSCT should be considered earlier in the treatment course.
